# Knowledge and Attitude toward Antibiotic Use and Identification of Financially Feasible Options to Curb the Spread of Antibiotics in Environment

**DOI:** 10.1155/2023/6403250

**Published:** 2023-12-11

**Authors:** Mayank Krishna, Nilesh Makwana, Ganesh S. Kakde, Sapna Puri, Arun S. Kharat

**Affiliations:** ^1^Department of Environmental Sciences, Kalindi College, University of Delhi, New Delhi 110008, India; ^2^Laboratory of Applied Microbiology, School of Life Sciences, Jawaharlal Nehru University, New Delhi 110067, India; ^3^Department of Biochemistry, Central University Haryana, Mahendragarh, Haryana, India; ^4^Ohio State University, Columbus, OH, USA

## Abstract

A survey on antibiotic literacy in terms of the use and abuse of antibiotics to track and understand antibiotic consumption is crucial to optimize the use of antibiotics and minimizing antimicrobial resistance (AMR). Purposive random sampling, using the snow-ball questionnaire technique, was adopted to ensure that the respondents distributed across India, coming from rural and urban settings, were adolescents as well as adults and had completed at least the higher secondary school level of education. Respondents were divided into five subcategories. The questionnaire was distributed between April 2021 and July 2021, during the second COVID-19 wave in India. The survey questionnaire included 34 questions, comprising multiple-choice and 5-point Likert scale-type questions. This study composed of 972 respondents. Most respondents considered antibiotics safe and frequently failed to discriminate between the symptoms of bacterial and viral infections, most often leading to self-prescription. About 34% of the rural participants and 50% of the urban participants considered antibiotic resistance a serious health concern. Antibiotic prescriptions by the medical or paramedical practitioner were largely empirical. At least 95% of participants acknowledged having heard about antibiotics; nearly 20% of antibiotic consumption came from nonprescription users, while 30% had not completed their antibiotic therapy for a variety of reasons. Sixty-two percent consumed antibiotics to treat cold and flu symptoms. Results from the survey suggest the presence of a crucial gap between the respondents' perception of antibiotics and levels of information regarding antibiotic use and misuse. The present study may serve as a benchmark that strongly recommends a financially feasible policy, which includes educating society regarding the spread of AMR and its severe consequences by incorporating AMR into the curriculum at the levels of senior secondary school and higher education.

## 1. Introduction

After the discovery of penicillin in 1929, a sharp rise was observed across the globe in the identification, production, and commercialization of antibiotics for the treatment of infectious diseases [1, 2]. The discovery of more than 150 antibiotics and semisynthetic derivatives was considered a panacea for infectious diseases caused by bacteria [3]. Even though antibiotic usage caused an initial drop in the levels of mortality and morbidity associated with common infections, the indiscriminate use of antibiotics exerted negative effects on the environment and human health. In fact, it is now considered one of the most pressing public health crises of the twenty-first century [4–8]. The overuse of antibiotics in human medicine and other commercial activities, for example, livestock production, poultry, aquaculture, and the food industry, has resulted in “antibiotic pollution” [9]. Antibiotics have an “afterlife” and remain biologically active even after being discharged into the environment. The influx of antibiotics into the environment has resulted in severe consequences at the levels of the individual, community, and ecosystem, and the antibiotic residues may trigger antibiotic resistance [10]. An individual who frequently consumes antibiotics may develop allergic reactions, microbiome dysbiosis, and antibiotic resistance within the body. Furthermore, human excretory waste and industrial discharge have resulted in the introduction of antibiotic-resistant bacteria into the soil and water. Over a period of time, these resistant clones become part of the food chain and reenter the human body via the crops and vegetables that are grown on these soils [10–14]. The presence of resistant bacteria in the microbiome raises the body's susceptibility to opportunistic infections. Consequently, due to infection caused by antibiotic-resistant bacteria, the death rate has escalated at an alarming rate over the last three decades [1, 6–8]. As a result, paradoxically, the drugs that revolutionized the medical world in the treatment of bacterial infections have now also increased the susceptibility of the individual to bacterial infections [1]. Furthermore, antibiotic overuse imposes economic and other intangible costs, as considerable expenditure is incurred for the treatment of resistant infections, resulting in extended hospital stays [15, 16].

To better understand the severity of the problems related to antibiotic overuse and the consequent antibiotic-resistant infections, the World Health Assembly, in its 2015 general meeting, drafted an action plan to address the problem. Various factors were identified that might have resulted in the indiscriminate use of antibiotics, including misperception among the patients and general public, self-medication, lack of adherence to the dosage regime, and advertisement and promotion by the pharmaceutical companies [17–19]. One of the objectives of the action plan suggested by the World Health Assembly to enhance societal knowledge about antibiotic resistance was through effective communication, education, and training [20–22]. In recent times, the Indian Council of Medical Research (ICMR), an apex body responsible for the formulation, coordination, and promotion of biomedical research in India, has identified *Enterococcus faecium*, *Staphylococcus aureus*, *Klebsiella pneumoniae*, *Acinetobacter baumannii*, *Pseudomonas aeruginosa*, and the Enterobacter species (ESKAPE), a group of nosocomial infection-causing bacteria, as those organisms responsible for the major antimicrobial resistance (AMR) concern in India.

The first step toward addressing the problems related to antibiotic misuse and correlated antibiotic resistance is to clearly comprehend the antibiotics, track the consumption patterns of these antibiotics, and gain insight into their use at an individual level [17, 18]. This will facilitate formulating policies and shaping public campaigns that may curb antibiotic misuse, ensure antimicrobial supervision, and reduce the antibiotic load on the environment.

The current study has investigated the degree of awareness and knowledge regarding antibiotic use and antibiotic resistance among the general public in India, a country considered one of the pharmaceutical hubs in the world, where self-prescription, empirical therapy (clinical settings), and uncontrolled industrial applications are the main drivers of antibiotic use in light of the drug inequity that prevails in Indian society. In India, for the most part, prescriptions for antibiotics are often experimental, which are finally found to be inappropriate; in fact, over 98% of the prescriptions are given without any knowledge of the etiological agents, with reference either to their nature or the antibiotic sensitivity of the patient. A survey of the antibiotic literacy and use and abuse of antibiotics—to track and understand their consumption patterns—is crucial in order to optimize antibiotic usage and thereby minimize antibiotic-resistant infections [23–28]. Keeping the adolescent and adult population as the respondents, in order to gain a comprehensive assessment of the degree of knowledge related to antibiotics and their use, the questionnaire used in the present work has been divided into the following four domains: A. Level of knowledge about antibiotics; B. Indications of antibiotic use or abuse; C. Knowledge about antibiotic resistance; D. Action toward antibiotic resistance.

## 2. Materials and Methods

**Figure 1:**
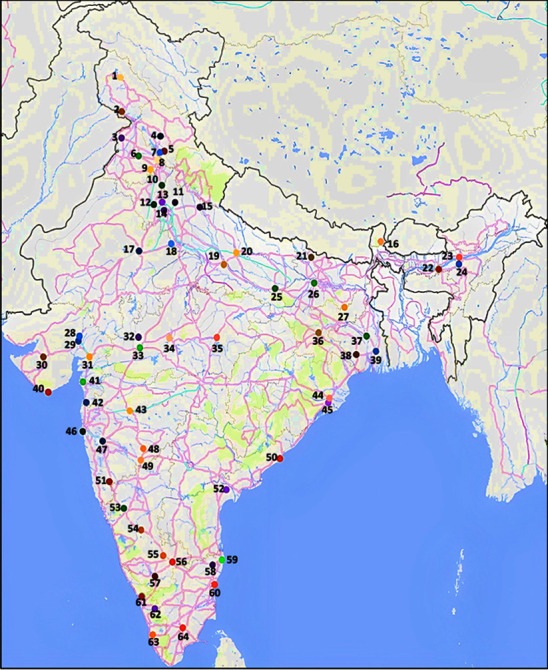
Schematic representation of various sampling sites (districts), which are mentioned as coloured dots with number: sixty-four districts extending across 21 states and three union territories in India (districts and union territories are shown below). (1) Srinagar, (2) Jammu, (3) Amritsar (4) Mandi, (5) Shimla, (6) Ludhiana (7) Solan, (8) Chandigarh, (9) Patiala, (10) Karnal, (11) Meerut, (12) Rohtak, (13) Sonipat, (14) Delhi, (15) Rampur, (16) Gangtok, (17) Jaipur, (18) Bharatpur, (19) Kanpur, (20) Lucknow, (21) Motihari, (22) Guwahati, (23) Tezpur, (24) Nagaon, (25) Varanasi, (26) Patna, (27) Deoghar, (28) Gandhinagar, (29) Ahmedabad, (30) Rajkot, (31) Vadodara, (32) Ujjain, (33) Indore, (34) Bhopal, (35) Jabalpur, (36) Ranchi, (37) Bardhman, (38) Medinipur, (39) Kolkata, (40) Diu, (41) Surat, (42) Dadra Nagar & Haveli, (43) Aurangabad, (44) Cuttack, (45) Bhubaneswar, (46) Mumbai, (47) Pune, (48) Osmanabad, (49) Solapur, (50) Vishakhapatnam, (51) Kolhapur, (52) Guntur, (53) Dharwad, (54) Davangere, (55) Tumkur, (56) Bengaluru, (57) Mysore, (58) Kanchipuram, (59) Chennai, (60) Puducherry, (61) Palakkad, (62) Kottayam, (63) Thiruvananthapuram, and (64) Madurai.

Respondents and study design: A self-reported questionnaire (Supplementary information 1) was developed to assess the knowledge and perception regarding antibiotics and antibiotic resistance among the adolescent and adult populations of India. Since the adolescent population plays a key role in the emergence and spread of bacterial resistance to antibiotics, the purposive sampling method using the snow-ball survey approach was adopted to ensure that all respondents were at least adolescents and those who have achieved a secondary or higher than the secondary level of education. The respondents were based in rural, suburban, and urban areas and spread across different regions of India. Furthermore, based on their educational levels, the respondents were divided into five categories, namely, (1) high schools; (2) senior colleges and universities; (3) corporates, (4) medical-paramedical-pharmaceuticals, and (5) NGOs dealing with environmental safety. The responses collected from the respondents were accomplished through a formal snow-ball survey approach. A questionnaire was disseminated through electronic invitation, requesting the respondents to provide their answers to the questions electronically, at their will. The questionnaire was distributed between April 2021 and July 2021, during the second COVID-19 wave in India. In fact, the survey consisted of 34 questions in total, comprising both multiple-choice and 5-point Likert Scale-type questions, distributed over four broader sections: A. Level of knowledge of antibiotics; B. Knowledge about antibiotic resistance; C. Mis(use) of antibiotics; and D. Action toward antibiotic resistance. The questionnaire was designed to help understand each respondent's perception with reference to the levels of information possessed regarding antibiotic use in self-medication. The survey included certain questions that served as internal controls to verify the authenticity of the responses obtained from respondents. All the responses received were coded and submitted to statistical analysis using SPSS version 21. Since antibiotic awareness was to be estimated, we selected respondents, ensuring that they possessed an educational level of completing high school at least. Adolescent students, parents, teachers, those with higher education, medical as well as paramedical students, teachers, along with the corporate and NGO service providers were included to ensure that most components of Indian society were participants in this study. Responses to the questionnaire were collected from across India, with the respondents' locations including the specific districts in India (Figure 1). We sought to collect at least 100 responses from each of the five categories. The number of respondents across India who provided answers to the questionnaire was 972. From among these 972, the high school category (student-parent and teachers) included 349, the senior college/university category had 288, the corporate sector showed 116, the medical and paramedical numbered 120, while the respondents from the NGO category were 100 in total.

## 3. Results

### 3.1. Overview of the Respondents' Profiles

Out of the 972 respondents, 637 (65.5%) were found to belong to the categories of senior secondary students, parents, teachers, and those from senior colleges and universities (Table 1). Male and female respondents were almost equal in number, with 665 (68.42%) respondents living in the suburban and urban areas and 267 (27.47%) in the rural areas. Approximately 71% of the total respondents had achieved secondary school-level education at least, and some had attained above this (Table 2). In terms of age groups, 80.35% of respondents fell within the range of 20–39 years of age.

The respondents' knowledge and attitudes toward antibiotics were assessed in this study, as well as their use and abuse of antibiotics, which could help to check the spread of antibiotic resistance in the environment.

### 3.2. Theme A: Level of Knowledge about Antibiotics

In the present survey, most respondents had heard about antibiotics, irrespective of their locality: rural respondents (95.13%) and urban respondents (96.69%). The major sources of their knowledge regarding antibiotics were books, the news, advertisements, and common knowledge (Table 3). In fact, the majority of the respondents, 539 (55.45%), could differentiate between antibiotics and other drugs; 55.43% were from the rural setting, while 56.54% were from the urban setting. Interestingly, of the 433 (44.55%) respondents, 44.73% were rural and 43.46% were urban and were unable to differentiate between antibiotics and other drugs (Table 3). The respondents from rural versus urban regions that were either able to or unable to discriminate between antibiotics and drugs were statistically insignificant (*t*-test, *p* < 0.5). The survey thus done suggested that the majority of the respondents (85.02% rural and 86.02% urban) used antibiotics prescribed by doctors. Most of the respondents (73.41% rural and 81.2% urban) agreed that different antibiotics were necessary to cure different diseases (Table 4). The difference observed between the rural and urban study populations after the *t*-test was done was found to be statistically significant (*p* < 0.05). From this study, it became evident that although the majority of respondents used antibiotics when prescribed by doctors, a sizable percentage of the respondents (approximately 20.61% from rural and 12.48% from the urban settings) had purchased the antibiotics from a pharmacy without any prescription. The responses from about 13.86% of rural and 7.8% urban areas indicated that self-prescription was done (Table 4).

### 3.3. Theme B: Knowledge regarding Antibiotic Resistance

**Figure 2:**
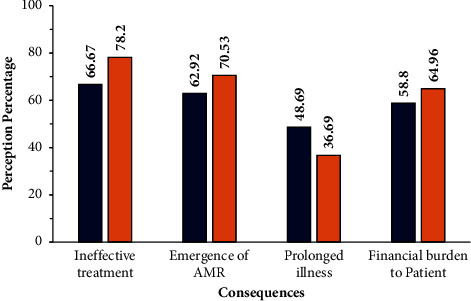
Respondents' perceptions on indiscriminate antibiotic use and the consequences. Four types of consequences due to indiscriminate antibiotic use are given. Values in the bar indicate the percentage of the respondents. The blue colour indicates the responses of the rural group, while brick red indicates the responses of the urban participants.

When queried about antibiotic resistance, the respondents related it to a condition that culminated in a state where the body became resistant to a particular antibiotic and understood that the specific antibiotic was no longer effective in controlling the infection 65.92% from rural areas and 70.68% from urban areas (Table 5). The reasons for developing antibiotic resistance were attributed to the overuse of antibiotics (63.67% rural and 45.51% urban), as well as to the incomplete course of an antibiotic. The difference noted among the rural versus urban respondents when tested with a *t*-test was found to be significant *p* < 0.05. Of interest, most of the respondents agreed that antibiotic resistance posed a serious public health issue across the world (65.54% rural and 39.7% urban) (*p* < 0.05), and was associated with individuals who took antibiotics on a regular basis (33.83% from rural and 45.57% urban settings) (Table 5). However, only a small percentage of respondents regarded antibiotic resistance as a serious health concern in India: 29.59% from rural settings and 18.05% from urban settings (Table 5). A significant percentage of the respondents (54.31% rural and 35.34% urban) agreed that the indiscriminate use of antibiotics culminated in ineffective treatment in the case of infections in the future, with prolonged illnesses placing a heavy financial burden on the patients and the emergence of antimicrobial-resistant genes in the environment (Figure 2).

### 3.4. Theme C: (Mis)use of Antibiotics

**Figure 3:**
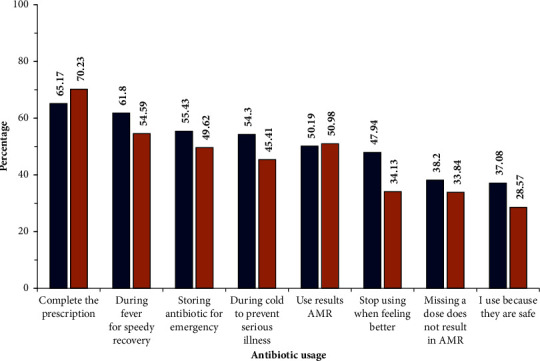
The perceptions of the participants in terms of safety, use, and the discontinuation of antibiotics are shown. The responses recorded to the questions pertaining to antibiotic use are given. The values in the histogram indicate the percentages of the respondents. The blue colour indicates the responses of the rural group, while brick red indicates the responses of the urban participants.

**Figure 4:**
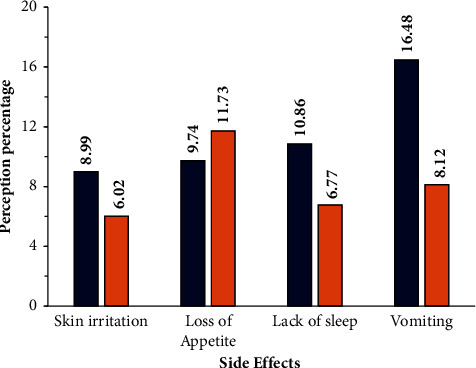
Responses on the side effects experienced after antibiotic use. Four types of side effects were reported by both the urban and rural respondents. The blue colour indicates the responses of the rural group, while brick red indicates the responses of the urban participants.

In order to recognize the latent drivers that cause the (mis)use of antibiotics at an individual level, the perception as well as the knowledge regarding antibiotic use needs to be evaluated. To accomplish this, the gap between perceptions related to antibiotic use and levels of knowledge was estimated by including questions that acted as internal controls. When questioned about their perceptions regarding antibiotic use, most of the respondents considered antibiotics to be safe drugs and believed they were the best choice for the rapid cure of a fever and prevention of any serious illness during a cold, according to the responses of 62% rural and 53% urban respondents (Figure 3). When this perception was analysed using the *t*-test, a significance of *p* < 0.05 was found. This was one reason the respondents stocked up on antibiotics for emergency use. Once they began to feel better, they stopped taking the antibiotics—the responses from 45% rural and 34% urban participants. They did not consider that missing a dose of the antibiotic could result in AMR—the responses of 38% rural and 33% urban participants (Figure 3). On analysis, no significance was found (*p* < 0.05). Most of the respondents associated the side effects of the antibiotics with mild symptoms like vomiting, lack of sleep, loss of appetite, and skin irritations (Figure 4).

**Figure 5:**
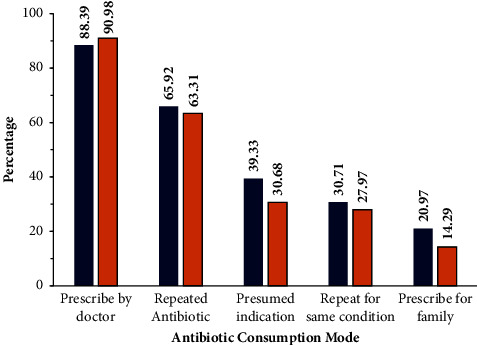
Sources and indications of antibiotic usage. Percentage values are shown in the histogram for the medical prescription to patient/family, repeating dose, repeating for the same condition, and presumed indications, . The blue colour indicates the responses of the rural group, while brick red indicates the responses of the urban participants.

**Figure 6:**
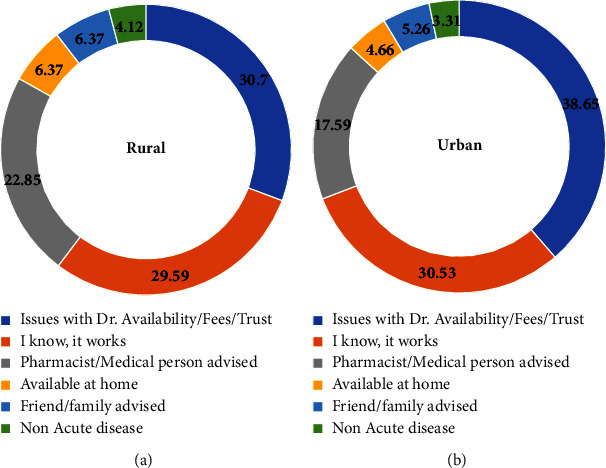
Reasons for self-medication. The responses against self-medication are shown. The values indicate the percentage of the respondents; the blue colour indicates various reasons related to the doctors; the orange denotes antibiotic usage because “I know it works”; the grey colour denotes usage after consulting a pharmacist/medical person; the yellow colour denotes usage because it was available at home; the navy blue denotes usage after a suggestion was made by a family member/friend, while the green colour indicates usage because the disease was nonacute in nature. (a) Rural. (b) Urban.

Although most of the respondents used antibiotics prescribed by doctors, they repeated the consumption of the same antibiotics (65.92% rural and 63.31% urban participants), and the differences seen were statistically insignificant (*p* < 0.05). Repeating the antibiotic for similar health conditions was evident in 30.71% of rural and 27.97% of urban participants (statistically insignificant *p* < 0.05) or as prescribed to other family members (responses of 20.97% rural and 14.29% urban participants) (Figure 5). Prescriptions by other family members were more evident among the rural respondents than the urban respondents, and when tested with the *t*-test, differences were found to be statistically insignificant; *p* < 0.05. The reasons attributed for the practice of self-prescription of antibiotics, in both rural and suburban/urban areas were doctor availability (according to 30.7% rural and 38.65% urban participants), prior knowledge regarding antibiotic use as an option for the treatment of certain symptoms (as responded by 29.59% rural and 30.53% urban participants), or as advised by pharmacists or medical persons other than doctors (according to 22.85% rural and 17.59% urban participants) (Figure 6). The different reasons for self-prescription among the rural versus urban participants when a *t*-test was done showed no significance, *p* < 0.05. While 30.7% of the rural and 38.65% of the urban participants practiced self-prescription, this habit was attributed to doctor availability and fees. Another reason was the patient attitude of “I know, it works” which was clearly observed in 29.59% of the rural and 30.53% of the urban participants and showed no statistically significant difference.

**Figure 7:**
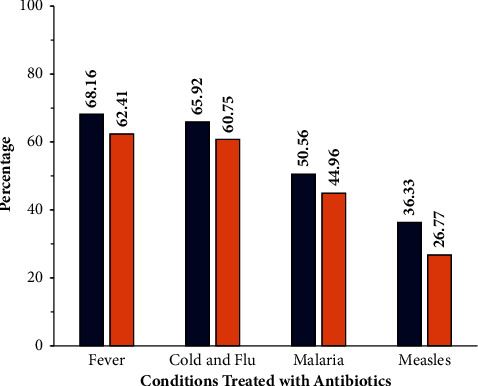
Perception that common symptoms can be treated with antibiotics. Values in the histogram show the participant responses, in percentage. The blue colour indicates the responses of the rural group, while brick red indicates the responses of the urban participants. Perception is for the belief that the common symptoms such as fever, cold, flu, malaria, and measles can be treated with antibiotics.

Interestingly, when asked about the common symptoms that could be treated with antibiotics, a large percentage of the respondents did not know that antibiotics were ineffective against the commonly occurring symptoms or syndromes, such as the common cold and flu (as responded by 65.92% rural and 60.75% urban participants), malaria (according to 50.56% rural and 44.96% urban participants), and measles (as responded by 36.33% rural and 26.77% urban participants), and equated viral infection with bacterial infection (Figure 7). Indiscriminate antibiotic use appeared more in the rural population than in the urban population, and when analysed using the *t*-test, it was found to be significant *p* < 0.05. A large percentage of the population even considered using antibiotics as a treatment choice for a sore throat (59.05%), headaches (30.45%), and body ache (32.10%), as well as skin and wound infections (52.56%) (Table 6). Of these symptoms, sore throat can be due to bacterial infection, allergic reaction, or a viral infection, infections can be monobacterial or polymicrobial and require microbial culture to ensure correct antibiotic therapy. Headaches, body aches, and some of the skin ailments are not necessarily bacterial.

### 3.5. Theme D: Action toward Antibiotic Resistance

A large percentage of respondents preferred traditional medicines/home remedies to treat the common symptoms (according to 70.04% rural and 68.27% urban participants) (Table 7). Total compliance with doctors' prescriptions was observed in a higher percentage among the urban respondents than among the rural respondents (65.71% in urban areas vs. 52.81% in rural areas). However, a large percentage of respondents had failed to complete the antibiotic dose regimen and prematurely discontinued the treatment when they observed improvement in their health condition (32.21% rural and 24.36% urban respondents) (Table 7). Noncompliance with doctors' prescriptions was found to be higher among the rural participants than among the urban participants, *p* < 0.05. This was significant, as more than 50% of the respondents, when asked about their perception toward antibiotic resistance, attributed an incomplete course and lower doses of antibiotics as the reasons for antibiotic resistance (Table 5).

## 4. Discussion

The first step toward addressing the hazards of antibiotic overuse and antimicrobial resistance in the environment is to evaluate the perception and knowledge of the general public regarding antibiotics, as well as their use/overuse and abuse [23, 24, 27]. As perception is the single most important driver that controls the attitude and behaviour of a human being, assessing it becomes crucial. Our survey, done for the first time in India, focused on the adolescent and adult populations, which is important because the chief goal of this work is to understand the perception and degree of knowledge regarding antibiotics and antimicrobial resistance among the fairly well-educated population with substantial exposure to the digital world and a great deal of free exchange of information [5, 9, 27, 29]. As earlier studies suggested, a strong association exists between the general level of education and attitudes toward antibiotic use [24]. Our aim was to investigate whether the same was true for India. To the best of our knowledge, this is the largest survey conducted in India, focused only upon adolescents and an adult population across various regions of the country. Purposive sampling using the snow-ball survey approach and comprising a very large sample size of respondents has enabled us to examine the gaps that exist between perception, knowledge, and prudent antibiotic use.

### 4.1. Antibiotic Knowledge and Antibiotic Resistance

Our results indicate that even as advertisements and common knowledge continue to rise, public knowledge regarding antibiotics among the educated class is also becoming counterproductive in terms of self-medication, a fact that concurs with the reports from earlier studies for adult consumers, adult Hispanic consumers, and health care providers in the United States [25]. The general knowledge and attitude of people toward antibiotics have been reviewed by Gualano et al. [26] and McNulty et al. [27]. Gualano et al., in their systematic review, recommended strengthening educational initiatives to push physicians to spread awareness and the importance of correct behaviour in communities concerning antibiotic usage. Questionnaire-based studies performed by McNulty et al. on the British population indicate that there is no direct relation between increased antibiotic knowledge and more prudent antibiotic use. The work reported here is a first attempt to estimate Indian society with reference to antibiotic knowledge, antibiotic resistance, use, and misuse, as well as actions to curb antibiotic resistance in the Indian set-up. In some European Union countries, for instance, Poland, and some North American countries, antibiotics can be obtained only through doctors' prescriptions [23–28]. Similar guidelines have been issued by the Indian Medical Association; however, it appears that their implementation in the country is impeded by a bottleneck. We discovered the absence of a straightforward relationship between the rise in knowledge and prudent antibiotic use, which concurs with the findings of earlier studies conducted by Abdelmalek et al. [30]. Since antibiotic knowledge and escalating antibiotic resistance did not reveal a straightforward relationship, it implied that other health concerns or problems were more serious than the health concerns induced by antibiotic resistance. Also, knowledge regarding the prevalence of antibiotic-resistant genes at the community level and in the environment was most likely caused by their indiscriminate use by individuals, which is evident from the responses of the respondents. A critical analysis of the responses of the respondents presented in Tables 4 and 5 reveals that, at present, the participants do not understand that they play a personal role in the attempts to control antibiotic resistance.

### 4.2. Mis(use) of Antibiotics

We found that although most respondents theoretically understand that antibiotics are used solely in the treatment of bacterial infections, they have often failed to discriminate between bacterial-induced symptoms and viral-induced symptoms, a finding that concurs with the studies reported earlier [24, 29]; this has often resulted in self-prescription, reiterating that the implementation of the IMA guidelines needs closer and more intense monitoring. Despite understanding that antibiotic overuse may result in serious health problems, it continues to remain in the backseat because, in the intellectual landscape, other health conditions dominate it. Even the general educated class of society seems to be oblivious to the concept that antibiotic loading in the environment is due to the overuse of antibiotics at the individual level. This corresponded to the previous reports, suggesting rather extensive ignorance regarding the ineffectiveness of antibiotics against viral infections as well as a lack of knowledge about the differences in the symptoms induced by bacteria and viruses [24]. Similar confusion was observed with respect to the use of antibiotics in the treatment of bacterial and viral infections in some studies reported from Sudan and Kuwait [29]. However, this may be a cause for concern for India because the respondents in our study were fairly well educated and a wide age range was covered, including both adolescents and adults. This reflected the presence of a major gap between perceptions and knowledge related to antibiotic use. Society needs to be responsible for the prevalent attitude of the population toward antibiotics, which has ultimately resulted in the over exposure, over prescription, and misuse of antibiotics.

One of the important aspects linked to the reduction in antibiotic resistance was correct dosage and adherence to a complete dose regimen of antibiotics as prescribed by the doctors. Our study revealed that despite the vast majority of respondents having completed the dose regimen per the doctor's prescriptions, there was still a considerable percentage of respondents (mostly from rural areas) who had discontinued it prematurely once they noticed improvement in their health conditions. Although they all agreed that the overuse of antibiotics could lead to antibiotic resistance, they were unaware of the association between an incomplete dose regimen and antibiotic resistance and considered antibiotics to be safe drugs. One method of spreading awareness would be incorporating the consequences of the misuse of antibiotics into the academic curriculum, from the secondary level of education itself up to the levels of higher education.

### 4.3. Action toward Antibiotic Resistance

Acceptance of traditional medicine and home remedies for the treatment of common symptoms is a positive sign that may facilitate curbing the rapid spread of antimicrobial resistance [31].

### 4.4. Financially Feasible Policy for Prudent Antibiotic Use

Our study suggests that in order to address the concern regarding self-medication, it becomes necessary to fill in the crucial gaps between perception and knowledge with a more targeted campaign, similar to the approach described by Mazińska et al. [28]. This may include the integration of antibiotic knowledge with reference to benefits versus consequences and their prudent use versus overuse, with the priority being medical personnel and pharmacists, so that the overprescription of antibiotics can be monitored and self-prescription curbed. Awareness regarding prudent antibiotic use and the ability to discriminate between bacterial and viral infections must be spread among the adult and adolescent populations through targeted campaigns and Citizen Science Programs in schools, colleges, and offices. This can also be achieved through popularization of science, which is the theme of the INSPIRE campaigns in the Department of Science and Technology, Government of India. There is an urgent need to convince the general public, through repeated messages, that in the case of less severe infections such as a cold and flu, adopting traditional medicine as an alternative to antibiotics is more effective in the long term and helps boost individual immunity as well—a finding that concurs with those described previously by Fazil and Nikhat [31]. Spreading awareness will require the active and constructive involvement of government institutions, the media, scientists, and researchers. Robust, effective, and repetitive communication to the general public regarding antibiotic use and antimicrobial resistance must be implemented.

#### 4.4.1. Global Antimicrobial Stewardship

Antimicrobial Stewardship (AMS) programs adhering to the Global Action Plan on AMR were established by the World Health Assembly in 2015; this is expected to enable clinicians to optimize the use of antimicrobials, raise patient outcomes, reduce AMR and healthcare-associated infections, and save healthcare costs, among other things, by improving the prescribing of antibiotics and building the capacity of healthcare professionals to the best possible extent in terms of the rational use of antibiotics [32–34]. The questionnaire employed in this study was intended to collect, analyse, and interpret data on the appropriate use and prescription-based antibiotic use in order to arrive at a conclusion about whether India is approaching the AMS mandate or needs to intensify the mandate. Several countries have now developed and are implementing National Action Plans (NAPs) on AMR, in which AMS is a key priority and is anticipated to play a pivotal role in curbing the emergence of AMR. In India, the Indian Council of Medical Research (ICMR) recognized the significance of AMS in healthcare and initiated the activities of the AMS program by developing the AMS program curriculum. In 2013, India initiated the Antimicrobial Resistance Research and Surveillance Network (AMRSN) to respond to AMR and thus ensure the rationalization of antimicrobial use. According to the AMRSN annual report, there has been an overall increase in the pattern of antimicrobial resistance to Enterobacterales and other pathogenic groups isolated from patients over the last five years, from 2017 to 2021 (Supplemental Figures 1A–1E) [35].

#### 4.4.2. Strategies for Antibiotic Usage among the Indian Population

India was one of the largest antibiotic consumers in the world because of the empirical antibiotic prescription—prescription without evidence of bacterial infections or based on nonbacterial evidence in OBG patients [36]. During the 2000–2010 decade, India revealed 23% antibiotic consumption out of 76% of the global antibiotic consumption. The rate of consumption, however, varied from state to state in India. From 2011 to 2019, the consumption rates declined, but with an increased inappropriate use of antibiotics in the high-focus states in comparison to the non-high-focus states of India [37]. Therefore, to improve global antibiotic usage, the WHO Expert Committee classified antibiotics on the EML (Essential Medicines List), which were subdivided into three categories under the AWaRe Classification (Access, Watch, and Reserve) [38]. It is essential that antibiotics are prescribed only when necessary and that the last-resort antibiotics come from the REserve groups of AWARE, to be availed only to needy patients. In India, mostly in the “Watch” group, antibiotics are prescribed, followed by the “Access” group of antibiotics [39]. The National Centre for Disease Control (NCDC) released national treatment guidelines for antimicrobial use for infectious diseases in 2016 to restrict the inappropriate use of antibiotics [40]. In India, strategies were made for the use of antibiotics for the REserve group (Supplemental Table 1). It is anticipated that antibiotics from the REserve group will be compulsorily “prescribed only after the indications are affirmed by the clinical microbiology reports [40]. The RESERVE group of antibiotics is to be issued to treat infections that exhibit failure to the other antibiotic groups for about 48–72 hours, to patients who are recognized as severely immunosuppressed or to those with infectious organisms that are pan-drug resistant, or if some other safer drug option has been ruled out in the culture report.

## 5. Conclusion

Although 95% of the respondents acknowledged having heard about antibiotics, nearly 20% of the antibiotic consumption came from nonprescription users, while 30% had not completed their antibiotic therapy for a variety of reasons. The presumed indication of antibiotic use was 35%; a significant percentage (62%) consumed antibiotics to treat cold and flu symptoms. Antibiotic stewardship, and awareness in particular, needs to be inculcated, and the dire need of the hour is to intensify public awareness on antibiotic use/misuse. As a financially feasible policy, the antibiotic concept, use, and misuse, and especially the way AMR develops, must be integrated into the curriculum of adolescents at the senior secondary school level and later reiterated at higher learning levels, with specific reference to the mechanisms of developing AMR and its dissemination from the hospital/farm set-up to several other environmental compartments.

## Figures and Tables

**Table 1:** Overview of respondents' profiles.

Categories		(*n* = 972)	
		*N*	%
Type of category	Senior secondary student, teacher, and parent	349	35.9
	Senior college and university	288	29.6
	Medical college, paramedical college, and healthcare	119	12.2
	Corporate	116	11.9
	NGO	100	10.3
	Total	972	100

Gender	Female	483	49.69
	Male	438	45.06
	Transgender	7	0.72
	No response	44	4.53

Age	<20	191	19.65
	20–39	619	63.68
	40–60	110	11.32
	60+	4	0.41
	No response	48	4.94

In this study, 972 participants in total from various categories took part and provided the responses. Types of categories: senior secondary school—student/teacher/parent; senior college and university—student/teacher/non-teaching; medical college, paramedical college and healthcare-associated—student/teacher/professionals; corporate—managerial/associate and technical; and NGO—office bearer/supporting staff; gender and age are cited.

**Table 2:** Overview of respondents' profiles.

Categories		(*n* = 972)	
		*N*	%
Educational qualification	Secondary level	172	17.7
	Graduate level	267	27.47
	Postgraduate level	251	25.82
	No response	282	29.01
	Total	972	100

Locality description	Rural	267	27.47
	Urban	665	68.42
	No response	40	4.12
	Total	972	100

The educational qualifications of the participants and the locality they reside in, whether urban or rural, are listed. Most participants received education above the secondary school level. Two-thirds of the participants came from rural areas, while one-third came from the rural locality.

**Table 3:** Level of knowledge on antibiotics: antibiotic knowledge, source, and ability to differentiate them from other drugs.

Subthemes		Total		Rural		Urban	
		*N*	%	*N*	%	*N*	%
Heard of antibiotics	Yes	927	95.37	254	95.13	643	96.69
	No	22	2.26	9	3.37	11	1.65
	Maybe	13	1.34	3	1.12	8	1.2
	Do not know	10	1.03	1	0.37	3	0.45

Source of knowledge	Books	476	48.97	155	58.05	307	46.17
	Common knowledge	578	59.46	204	76.4	365	54.88
	Advertisement	205	21.09	63	23.6	131	19.7

Able to differentiate between antibiotics and other drugs	Yes	539	55.45	148	55.43	376	56.54
	Cannot differentiate	433	44.55	119	44.73	289	43.46

Responses were collected from 972 participants, and the response against each possible answer is estimated as the total percentage, separately for the urban and rural localities.

**Table 4:** Level of knowledge on antibiotics.

Subthemes		Rural		Urban	
		*N*	%	*N*	%
Who prescribed the antibiotic?	Doctor	227	85.02	572	86.02
	Paramedic	55	20.61	83	12.48
	Self	37	13.86	52	7.8

Belief that different antibiotics are needed to cure different diseases	Agree	196	73.41	540	81.2
	Neutral	52	19.48	97	14.59
	Disagree	19	7.12	28	4.21

Responses against antibiotic prescription and different antibiotic cures for different diseases are shown. All the responses of the 972 participants included in this study were recorded separately under the categories, rural and urban.

**Table 5:** Knowledge about antibiotic resistance.

Respondents' perceptions: causes of antibiotic resistance	Rural %	Urban %
Body becomes resistant to antibiotics, which no longer work on the infection	65.92	70.68
Wrong indication/lower doses	54.31	35.34
Overuse of antibiotics	63.67	45.51

*Respondents' perception: antibiotic resistance-affected components*		
Is it an issue for those who take antibiotics on a regular basis?	33.83	47.57
Is it a global/serious public health issue?	65.54	39.7
Is it a problem for our country—India—and not for other countries?	29.59	18.05

The perception of the participants on what triggers antibiotic resistance and how antibiotic resistance affected various components, is shown. Against each query, a record of the responses from the rural and urban participants is given, in percentage.

**Table 6:** Peoples' perceptions: antibiotics can cure (antibiotic use/abuse).

Perception: Antibiotics can cure		
Subtheme	*n*	%
Cold and flu	595	61.21
Malaria	444	45.68
Measles	277	28.50
Sore throat	574	59.05
Headaches	296	30.45
Body aches	312	32.10
Skin or wound infection	508	52.26

The perception by the participants that antibiotics can cure viral and parasitic diseases, as well as conditions that might arise from infection and noninfection, are shown. Only the responses indicating “yes” are scored in percentage.

**Table 7:** Action towards antibiotic resistance.

Subthemes		Rural	Urban	Total
Prefer traditional medicine/home remedies/allopathic	Yes	70.04	68.27	67.90
	No	28.84	31.43	31.58
	Do not know	1.12	0.30	0.51
	Total %	100	100	100

How did you stop an antibiotic course?	Upon improvement	32.21	24.36	26.03
	100% compliance with doctor's prescription	52.81	65.71	61.73
	After exhaustion	5.24	2.41	3.09
	Cannot remember	9.74	7.52	9.15
	Total %	100	100	100

The preferences of the participants toward the utilization or discontinuation of antibiotics are shown, in percentage, for the entire number of 972 participants, both urban and rural. The values in the table represent the participants' responses, in percentage.

## Data Availability

The relevant data will be made available at a later date upon request.

## Supplementary Materials

Supplementary Materials Supplementary Information 1: Questionnaire Form-ICSSR. Supplemental Figure 1: five-year susceptibility trend of (a) Enterobacterales isolated from all the samples, except urine and feces; (b) *A. baumannii* isolated from all the samples; (c) *Pseudomonas aeruginosa* isolated from all the samples; (d) *Burkholderia cepacia* isolated from all the samples; (e) *Stenotrophomonas maltophilia* isolated from all the samples. Supplemental Table 1: comparative statements, regarding the AWaRe-classified antibiotics, between the WHO and NCDS, India. Antibiotics prescribed for Empirical (Access), under the Watch Category and REserve, to be prescribed only to the needy patients, upon clinical microbiology indications.
