# Supplementary material for: Knowledge and Attitude toward Antibiotic Use and Identification of Financially Feasible Options to Curb the Spread of Antibiotics in Environment
Source: Can J Infect Dis Med Microbiol. 2023 Dec 11;2023:6403250. doi: 10.1155/2023/6403250 (PMC10728364; doi:10.1155/2023/6403250)
Supplement: Supplementary Materials — Supplementary Information 1: Questionnaire Form-ICSSR. Supplemental Figure 1: five-year susceptibility trend of (a) Enterobacterales isolated from all the samples, except urine and feces; (b) A. baumannii isolated from all the samples; (c) Pseudomonas aeruginosa isolated from all the samples; (d) Burkholderia cepacia isolated from all the samples; (e) Stenotrophomonas maltophilia isolated from all the samples. Supplemental Table 1: comparative statements, regarding the AWaRe-classified antibiotics, between the WHO and NCDS, India. Antibiotics prescribed for Empirical (Access), under the Watch Category and REserve, to be prescribed only to the needy patients, upon clinical microbiology indications. [file 6403250.f1.zip › Supplemental Table 1 CJIDMM.docx]

Supplemental Table 1: Table: Comparative analysis of AWaRe antibiotics between WHO and NCDS (India).

| WHO AWaRe Classification | | NCDS Antibiotic Use Classification | |
| --- | --- | --- | --- |
| Access Group | Amikacin,Amoxycillin, Amoxycillin + Clavulanic Acid, Ampicillin, Benzathine Benzylpenicillin, Benzylpenicillin, Cefalexin, Cefazolin, Chloramphenicol, Clindamycin, Cloxacillin, Doxycycline, Gentamicin, Metronidazole, Nitrofurantoin, Phenoxymethylpenicillin, Procaine benzylpenicillin, Spectinomycin, Sulfamethoxazole + Trimethoprim | Access Group | Amikacin^†^, Amoxycillin^†^, Amoxycillin +Clavulanic Acid^†^, Ampicillin^†^, Cefazolin^†^, Gentamicin^†^, Metronidazole^†^, Sulfamethoxazole + Trimethoprim^†^ |
| Watch  Group | Azithromycin, Cefixime, Cefotaxime, Ceftazidime, Ceftriaxone, Cefuroxime, Ciprofloxacin, Clarithromycin, Meropenem, Piperacillin + Tazobactam, Vancomycin | Watch  Group | Ciprofloxacin, Ceftazidime, Cefotaxime, Ceftriaxone, Vancomycin or Teicoplanin, Linezolid (Oral/IV), Imipenem, Levofloxacin, Meropenem, Moxifloxacin, Piperacillin + Tazobactam, Caspofungin, Valganciclovir, Azithromycin^†^, Cefixime^†^, Cefoperazone combination^†^, Ofloxacin^†^, Voriconazole, Ertapenem |
| REserve Group | Ceftazidime + Avibactam, Colisin, Fosfomycin (IV), Meropenem + Vaborbactam, Plazomicin, Polymyxin B, Linezolid | REserve  Group | Carbapenems, Linezolid, Colistin, Rifampicin, Aminoglycosides |

^†^Antibiotic list from Hisa *et al.,* 2019.
