# Supplementary material for: Knowledge and Attitude toward Antibiotic Use and Identification of Financially Feasible Options to Curb the Spread of Antibiotics in Environment
Source: Can J Infect Dis Med Microbiol. 2023 Dec 11;2023:6403250. doi: 10.1155/2023/6403250 (PMC10728364; doi:10.1155/2023/6403250)
Supplement: Supplementary Materials — Supplementary Information 1: Questionnaire Form-ICSSR. Supplemental Figure 1: five-year susceptibility trend of (a) Enterobacterales isolated from all the samples, except urine and feces; (b) A. baumannii isolated from all the samples; (c) Pseudomonas aeruginosa isolated from all the samples; (d) Burkholderia cepacia isolated from all the samples; (e) Stenotrophomonas maltophilia isolated from all the samples. Supplemental Table 1: comparative statements, regarding the AWaRe-classified antibiotics, between the WHO and NCDS, India. Antibiotics prescribed for Empirical (Access), under the Watch Category and REserve, to be prescribed only to the needy patients, upon clinical microbiology indications. [file 6403250.f1.zip › Supplementary information 1 Questionnaire.docx]

Questionnaire Form- ICSSR

**Studies on Identifying the Financially Feasible Policies to**

**Reduce the Spread of Antibiotic Resistance in Environment**

**NOTE: Remove Not application option(s). Choose an appropriate option, make it bold and send it back to the same e-mail you received this questionnaire.**

The project aims to comprehend the perception, knowledge about the irrational use of antibiotics and sensitization at the community level about the adverse effects and consequences of self-medication practice. This questionnaire will help us understand participants’ perception and levels of information about the antibiotic use, consequences of self-medication. This would help us in streamlining the standard guidelines for the antibiotic use. Your participation will involve completing a questionnaire that will take around 15 minutes. There are no anticipated risks by participating in this project. The information you provide will only be used as part of the project. However, to ensure the confidentiality, your name is not required while responding questionnaire. Your participation is totally voluntary and you can withdraw from this study at any time.

**Informed consent to participate:** I voluntarily agree to participate in the research by completing the questionnaire:

1. Yes
2. No
3. **Documenting the knowledge of antibiotics**
4. Have you ever heard of Antibiotics?
   1. Yes
   2. No
   3. Not sure
5. Source of knowledge/information about antibiotic
   1. News/media
   2. Books
   3. Common parlance
   4. Gathered from group discussion (s)
   5. Advertisement (s) at government portals/Health centers
6. Do you visit a family doctor or chose at random for your illness/health conditions?
7. Common medicine (s) you keep as the first aid?
8. Have you ever used any Antibiotic prescribed by the doctor?
   1. Yes
   2. No
9. If **Yes**, then which illness/ symptom /infection was treated with the antibiotics? (*Select more than one option*) (at the scale of 0-5)
   1. Fever
   2. Common cold and cough
   3. Diarrhea
   4. Sore throat
   5. Urine infection
   6. Pneumonia
   7. Gastrointestinal tract diseases /stomach disturbance
   8. Respiratory diseases
   9. Other (please specify)
   10. Do not remember for which condition
10. Have you ever repeated the antibiotic for the same health condition for which you were earlier prescribed by your doctor?
    - 1. Yes
      2. No
11. Have you ever used antibiotics without a prescription for the presumed indication?
    1. Yes
    2. No

5. Do you know about bacteria that is good for human health? (Y/N)

6. Does antibiotic negatively affect useful bacteria also?

1. **Information assessing the use of Antibiotics**
2. When did you take last antibiotics?
   1. 0-3 months before
   2. 3-6 months
   3. > 1 year
   4. Can’t recall or don’t remember
3. What was the reason for last antibiotic you took? _________________
4. Who prescribed antibiotic for you?
   1. Doctor
   2. Nurse
   3. Compounder
   4. Pharmacist/ Medical Store
   5. Self
5. Did you get an advice from a doctor, nurse or a pharmacist on how to use antibiotics?
   1. Yes, I was advised on how to take them (e.g., with food, on an empty stomach, after meal, once a day/ twice a day/ thrice a day & for 3 days/ for 5 days/for 7 days)
   2. No, there was no advise given in this regard
   3. Can’t remember
6. Where did you get these antibiotics?
   1. Medical store or pharmacy
   2. Hospital Dispensary
   3. Over internet/online platforms
   4. Friend or family member (Left over / Unused)
   5. I had them saved up from a previous time (Left over /Unused or kept as a contingency)
   6. Can’t remember
7. Can you differentiate prescription with reference to antibiotic and other drugs?
   1. Yes
   2. Can’t differentiate
   3. Dr. marked them separately
   4. It does not matter what cures, an antibiotic or another drug
8. Do you read the label information and indication of antibiotics before taking it? Yes/No
9. Do you drink alcohol while following the course (5-7 days) of antibiotics? Yes/No
10. **Information assessing the knowledge about Antibiotics Use**
11. To what extent you agree or disagree that it’s okay to use those antibiotics which were prescribed to a friend or family member suffering with the same illness/condition/symptoms that you are suffering with, at present?
    1. Strongly agree
    2. Agree
    3. Neutral
    4. Disagree
    5. Strongly disagree
12. To what extent you agree or disagree to buy the same antibiotics, or request for the same from your doctor, every time you are sick with similar symptoms?
    1. Strongly Agree
    2. Agree
    3. Neutral
    4. Disagree
    5. Strongly Disagree
13. To what extent you agree or disagree that different antibiotics are needed to cure different diseases?
    1. Strongly Agree
    2. Agree
    3. Neutral
    4. Disagree
    5. Strongly Disagree
14. How did you stop your antibiotic course?
    1. Upon improvement of symptoms or feeling better –non compliance
    2. 100% compliance with Dr. prescription
    3. After exhausting my purchased quantity –non compliance
    4. Can’t recollect
15. If you self-medicate (bought antibiotic without Dr. prescription) then what triggered you to consume antibiotic without a medical prescription?
    1. I know it works for the symptoms
    2. It was available at home
    3. Friend / family member advised
    4. Pharmacist /medical store advised
    5. Disease symptoms were not as acute (less painful)
    6. To reduce Dr. fees –it’s expensive otherwise
    7. Can’t wait for medical appointment
    8. Afraid of hospitals and Dr.
    9. No Dr. in the vicinity
    10. Some distrust among Dr.
    11. Don’t know
16. Who motivated you to take the medication without prescription?
    1. Friends
    2. Parents/ spouse
    3. Family members
    4. Relatives /elders
    5. Your pharmacist or a person in the pharmacy

19. Have you ever faced side-effects due to antibiotic?

20. Which side-effects you had after consuming antibiotic(s)

A. Loss in appetite

B. Lack of sleep

C. Skin irritation

D. Vomit/bowel disorders

E. Any specific experience

1. Please answer whether you think these conditions can be treated with antibiotics using the scale.

| Sr. No. | Conditions | Treated | Non-treated | Unsure |
| --- | --- | --- | --- | --- |
| 01 | HIV/AIDS |  |  |  |
| 02 | Respiratory Infection / Pneumonia |  |  |  |
| 03 | Urinary Bladder Infection |  |  |  |
| 04 | Urinary Tract Infection |  |  |  |
| 05 | Diarrhea |  |  |  |
| 06 | Gonorrhea |  |  |  |
| 07 | Cold and flu |  |  |  |
| 08 | Fever |  |  |  |
| 09 | Malaria |  |  |  |
| 10 | Measles |  |  |  |
| 11 | Skin or wound infection |  |  |  |
| 12 | Sore throat |  |  |  |
| 13 | Body aches |  |  |  |
| 14 | Headaches |  |  |  |
| 15 | Covid-19 |  |  |  |

1. Will you prefer traditional medicine/ home remedies or/ allopathic medicine for the symptoms/disease for which you were prescribed/consumed antibiotics? (Y/N)
2. **Information assessing the knowledge about Antibiotic Resistance**
3. Please state ‘Yes’ or ‘No’ in regards to your knowledge on the following terms:

Have you ever heard about the terms mentioned below? Please mark as “Yes” or “No”.

| Sr. No. | Terms | Yes | No |
| --- | --- | --- | --- |
| 01 | Antibiotic resistant bacteria |  |  |
| 02 | Antimicrobial resistance -AMR |  |  |
| 03 | Multiple Drug Resistance -MDR |  |  |
| 04 | Extensively Drug Resistance –XDR |  |  |
| 05 | Pan Drug Resistance –PDR |  |  |
| 06 | Superbugs |  |  |

1. If your answer is ‘Yes’ to the first term of previous question, then when and where did you hear the term ‘Antibiotic Resistance’ for the first time?

1. Doctor or nurse/ hospital

2. Pharmacist/ medical Store

3. Family member or friend (including on social media)

4. Media (newspaper, TV, radio)

5. Specific campaign

6. Other

7. Can’t remember

25. Please indicate whether you believe that the following statements are true or false.

| Sr. No. | Terms | Yes | No |
| --- | --- | --- | --- |
| 01 | Antibiotic resistance occurs when the body becomes resistant to antibodies and they no longer work on the infections. |  |  |
| 02 | When antibiotics are taken for the wrong indication such as incomplete course or lower doses, it can lead to antibiotic resistance |  |  |
| 03 | Overuse of antibiotics can cause antibiotic resistance |  |  |
| 04 | Bacteria which are resistant to antibiotics can be spread from person to person |  |  |
| 05 | Indiscriminate and Injudicious use of antibiotics can lead to: |  | |
|  | a) Ineffective treatment |  |  |
|  | b) Increased A/E |  |  |
|  | c) Exacerbation or Prolongation of illness |  |  |
|  | d) Emergence of bacterial resistance |  |  |
|  | e) Additional burden of medical cost to the patient |  |  |
| 06 | Antibiotic resistance is only an issue for those who take antibiotics on a regular basis. |  |  |
| 07 | Antibiotic resistance is a global wide important and serious public health issue. |  |  |
| 08 | Antibiotic resistance is a problem for our country-India, but not other countries. |  |  |
| 09 | We should minimize antibiotics use as we have traditional home remedies available |  |  |

1. To what extent you agree or disagree with the following actions, which would help address the problem of antibiotic resistance in the following manner? *(Please select only one option)*

| Strongly Agree | SA |
| --- | --- |
| Agree | A |
| Neutral | N |
| Disagree | D |
| Strongly Disagree | SD |

| Sr. No. | Actions | (SA) | (A) | (N) | (D) | (SD) |
| --- | --- | --- | --- | --- | --- | --- |
| 01. | People should use antibiotics only when they are prescribed by a doctor or nurse |  |  |  |  |  |
| 02. | Farmers should substantially cut down antibiotics use in animal feed |  |  |  |  |  |
| 03. | People should not keep antibiotics and use them later for other illnesses |  |  |  |  |  |
| 04. | Parents should make sure all of their children’s vaccinations are up-to-date |  |  |  |  |  |
| 05. | Doctors should only prescribe antibiotics when they are needed |  |  |  |  |  |
| 06. | Governments should reward the development of new antibiotics |  |  |  |  |  |
| 07. | People should wash their hands regularly |  |  |  |  |  |
| 08. | Pharmaceutical companies should develop new antibiotics |  |  |  |  |  |

1. **Information assessing the belief and perception about the Medicine**
2. To what extent do you agree or disagree that medicines are beneficial for health?
   1. Strongly Agree
   2. Agree
   3. Neutral /Uncertain
   4. Disagree
   5. Strongly Disagree
3. To what extent do you agree or disagree that medicines are harmful for health?
   1. Strongly Agree
   2. Agree
   3. Neutral /Uncertain
   4. Disagree
   5. Strongly Disagree
4. To what extent do you agree or disagree that medicines are used more than required?
   1. Strongly Agree
   2. Agree
   3. Neutral /Uncertain
   4. Disagree
   5. Strongly Disagree
5. **Information assessing the attitude towards the Antibiotic Use**
6. Please carefully read each statement given and indicate your response for each item in the following manner:

| Strongly Agree | SA |
| --- | --- |
| Agree | A |
| Neutral | N |
| Disagree | D |
| Strongly Disagree | SD |

| Sr. No. | Statements | (SA) | (A) | (N) | (D) | (SD) |
| --- | --- | --- | --- | --- | --- | --- |
| 01 | When I have a cold, I should take antibiotics to prevent getting a more serious illness. |  |  |  |  |  |
| 02 | When I get fever, antibiotics help me to get better more quickly. |  |  |  |  |  |
| 03 | I always complete the full course of antibiotic treatment even upon the improvement of my symptoms. |  |  |  |  |  |
| 04 | I prefer to store up some antibiotics at home in case of emergency. |  |  |  |  |  |
| 05 | I read the instructions and labels of the antibiotic carefully. |  |  |  |  |  |
| 06 | Whenever I take an antibiotic, I contribute to the development of antibiotic resistance. |  |  |  |  |  |
| 07 | Skipping one or two doses does not contribute to the development of antibiotic resistance. |  |  |  |  |  |
| 08 | Antibiotics are safe drugs, hence they can be commonly used. |  |  |  |  |  |
| 09 | I stop taking the prescribed antibiotic once I feel better. |  |  |  |  |  |
| 10 | Antibiotic use should be restricted. |  |  |  |  |  |
| 11 | In my community, it is common to use antibiotics without a prescription. |  |  |  |  |  |
| 12 | Antibiotic may have negative side-effects. |  |  |  |  |  |

1. **Demographic Profile of the Respondent**
2. What is best description for your locality?
   1. Rural
   2. Urban
   3. Semi urban
3. What type of Medical Facilities are there in/ around your locality?
   1. Primary Health Care Center
   2. Sub-district hospital
   3. District civil hospital
   4. Private hospital
   5. Multi-specialty hospital
   6. Traditional medicine
   7. Herbal medicine
   8. Home remedies
   9. No facilities
4. Gender of the respondent
   1. Male
   2. Female
   3. Transgender
5. Age of the respondent (approximately in figures/numbers like 25 years old)…………...
6. Highest qualification of the respondent:
   1. Secondary
   2. Senior secondary school
   3. Para-medical school
   4. Medical college / institute
   5. Graduation in Science
   6. Graduation in other than science
   7. PG and Higher in Science /medical
   8. PG and Higher in other than science

1. Marital status of the respondent?
   1. Single
   2. Married
   3. Other
2. Do you have a health insurance?
   1. Yes
   2. No
   3. Not Aware about
3. If ‘Yes’ to the previous question, then which of the following?
   1. Public health insurance
   2. Private health insurance
   3. Both private and public insurance
